# Eating disorders in the time of COVID-19

**DOI:** 10.1186/s40337-020-00295-3

**Published:** 2020-04-20

**Authors:** Stephen Touyz, Hubert Lacey, Phillipa Hay

**Affiliations:** 1 School of Psychology and Inside Out Institute, University of Sydney, Sydney, New South Wales Australia; 2 St George’s Hospital, University of London, London, UK; 3 Schoen, London, UK; 4 Translational Health Research Institute (THRI), School of Medicine, Western Sydney University, Campbelltown, Australia; 5 Campbelltown Hospital, SWSLHD, Campbelltown, Australia

We have all been stunned by the speed of this viral pandemic. At the time of writing, one fifth of the world is under lockdown. The main foci have been on the public health effort to contain the spread of the virus, and the care of individuals with acute infection. We, in eating disorders, must have a broader brief. Not only must we help care for those sufferers who contract COVID-19, we must also address the impact –psychological, financial and social - on those that do not. The peculiarities of COVID-19 and the reaction of the public and governments to it, have particular relevance for people living with an eating disorder and those who care for them.

Traumatic events impact on people’s mental health. The fear of contagion and of the death of family members has created huge uncertainty. Isolation brings anxiety, sadness, anger and loneliness. Social distancing and ‘quarantine’ are against human nature. The negative emotional effects of “quarantine” [1] are likely to be accentuated for many anorexia nervosa sufferers who are already isolated both emotionally and physically. Impoverished interpersonal function will be more difficult to handle when severe “social distancing” is in place. These concerns do not just apply to people with eating disorders. Andrew Cuomo, Governor of New York, has said “People are struggling with the emotions as much as they are struggling with the economics” [2].

People with an eating disorder have a complex problematic relationship with food which will be enhanced at this time of food insecurity and panic buying. There will no doubt be a plethora of research in the coming months and years documenting what impact COVID-19 will have on the eating disorder community from both a clinician and patient perspective. Before speculating on these potential risks and outcomes, it is worth learning from past similar outbreaks and, where indicated, applying these efforts to the current COVID-19 pandemic. Data involving patients suffering from MERS, SARS, influenza and Ebola [3] were unequivocal in high risk populations (both healthcare providers and patients alike) in revealing a relationship between the neuropsychiatric symptoms experienced, and the outbreak concerned. There are similarities between these past outbreaks and the COVID-19 pandemic in that such outbreaks resulted in an ever-increasing sense of foreboding and fear, as well as elevated feelings of anxiety and panic and symptoms associated with post-traumatic stress disorder. What seems to be even more concerning is that there is now evidence to suggest that these adverse cognitive and psychiatric sequelae may have long lasting effects on people at risk [3].

The adverse impact that COVID-19 may have on the eating disorder population is at this stage unknown. However, there are some features that stand out and are deserving of our immediate attention. In the short term, the prevalence of COVID-19 will mean that standard treatment approaches should be re-considered. Should people who are undernourished and with cardio-vascular compromise, be admitted? Although there is some debate as to the medical risks associated with this before COVID-19, do these become more of a concern now? Will the number of admissions decrease during the pandemic? On the other hand, because of the fear of community transmission, would there be a feeling of safety with admission to an eating disorder programme, thus increasing admissions and placing an increased demand on such facilities.

The continuation of day hospital programmes for eating disorder patients during the pandemic raises many vexed issues including one of viability during this time. Because of physical distancing and the mantra around the globe of “staying at home” the running of face to face programmes becomes at the least challenging. There is ample evidence of cognitive behavioural therapy (CBT) group programmes being conducted utilising video conferencing (telehealth) with good clinical efficacy [4]. However, their adaptation to half day and full day programmes have yet to be investigated. The extension of online and alternate methods of delivering care from brief guided self-help CBT ([e.g. [5]) to more comprehensive care is urgently needed.

Many of our patients have rigid and inflexible eating behaviours with a very small range of foods that they can eat, and these are often brand related. At a time of food insecurity with bare supermarket shelves, brands may not be available resulting in less choice and increased risk of precipitous weight loss. The increasing news streaming regarding food insecurity may well act as a trigger for those who are ‘hoarders’ of food.

Very low weight people with anorexia nervosa may be particularly vulnerable to COVID-19 because of emaciation and their compromised physical health, although it isn’t clear that the degree that this applies to those less physically compromised. Studies have suggested that anorexia nervosa may accord a level of resilience to viral illness [6]. It is unclear why, but it could be that there are also non-biological effects, e.g. one explained by the social distancing experienced by people with this eating disorder. Notwithstanding this, if they do contract COVID-19 the effect is likely to be more profound than might be expected than for others of a similarly young age group.

Many people with bulimia nervosa and binge eating disorder are now at home for 24 hours a  day seven days per week. There is no escape from distancing oneself from food at home and there are limited opportunities to leave home to buy food. Bingeing on the family’s food when restocking is problematic, may lead to further family conflict, heightened emotional arousal, depression and anxiety as well as the likelihood of increased self- harm or even suicidality. Public Health England, a NHS body, and members of the British royal family released a set of guidelines on ‘mental health and well-being aspects of coronavirus’. [7]. Italy has reported suicides amongst medical staff and family members caring for COVID–19 sufferers because they felt responsible for the deaths [8]. Society will expect mental health professionals to address this.

As of this month, 10 million Americans had lost their jobs with limited or no income or medical insurance [9]. Episodes of binge eating can be extremely costly and at times of severe fiscal restraint can lead to theft of food and if caught the resultant compounding effects of having to deal with judicial system.

A former Australian of the Year and leading Psychiatrist, Professor McGorry, has made a call for the Australian government to urgently establish a national mental health response to address the increased need of the community at large [10]. We would go further and say an international response is needed to a virus which does not think nationally. Both the short-term and long -term consequences of having both an eating disorder and COVID-19 simultaneously are unknown and with time this is likely to become more apparent. It is therefore important that we rapidly develop a repository of comments, protocols, case histories, pertinent literature reviews as well as empirical papers on this topic. To expedite this, the Journal of Eating Disorders is running a special issue on this topic. There are, without doubt, many more important aspects warranting our immediate attention. We hope that this brief editorial will spur you, readers and researchers, into action.

## Data Availability

Not applicable.
